# Correlation Between Adjuvant Chemotherapy Regimen, Recurrence Pattern and Prognosis of Cholangiocarcinoma After Radical Surgery

**DOI:** 10.3389/fonc.2022.695228

**Published:** 2022-02-11

**Authors:** Le Rao, Na Ma, Yanli Liu, Lehui Du, Baolin Qu

**Affiliations:** Department of Radiotherapy, The First Medical Center of Chinese People’s Liberation Army (PLA) General Hospital, Beijing, China

**Keywords:** cholangiocarcinoma, radical surgery, adjuvant chemotherapy, recurrence pattern, prognosis analysis

## Abstract

**Background and Purpose:**

About 70% of patients with radical surgery Cholangiocarcinoma (CCA) have recurrence and metastasis. There are few studies on the relationship between CCA adjuvant chemotherapy (mono or combined therapy), recurrence pattern (local, regional, distant recurrence) and prognosis [(Disease free survival, DFS), (Overall survival, OS)] after radical surgery. This study focuses on the correlation between CCA adjuvant chemotherapy, recurrence pattern and prognosis.

**Methods:**

The study involved retrospective analysis of data: preoperative hematology, clinical pathology, adjuvant chemotherapy regimens, recurrence pattern, DFS and OS, of 207 patients with CCA. Chi-square test was used to analyze the correlation between related factors and postoperative recurrence. Survival curves were plotted by Kaplan-Meier method, P-values were calculated by Log-rank for univariate analysis, multivariate COX regression method for multivariate analysis.

**Results:**

Using chi-square test, there were correlations between high carbohydrate antigen 19-9 level(CA19-9≥35), vascular invasion, single-agent adjuvant chemotherapy and postoperative recurrences (p=0.04, p=0.04, p=0.02), COX multivariate regression analysis showed that adjuvant chemotherapy (single vs. doublet drug regimen) was an independent prognostic factor for DFS (11.0 vs. 24.6 months, HR=2.88, P=0.01), whereas recurrence pattern (local vs. distant; regional vs. distant) was an independent prognostic factor for OS (31.2 months vs. 20.4 months, HR=0.58, p=0.01; 32.0 months vs. 20.4 months, HR=0.51, p=0.01).

**Conclusion:**

Adjuvant chemotherapy regimen was an independent prognostic factor of DFS, whereas recurrence patterns were independent prognostic factors for OS. adjuvant chemotherapy with doublet drug regimen was correlated with longer DFS, and different recurrence modes affect OS.

## Introduction

According to the 2015 China Cancer Data Report, the number of new liver cancer patients and deaths was 466,100 and 422,100, respectively (1), accounting for 50% of the total number of liver cancer cases and deaths worldwide (2). Primary liver cancer includes hepatocellular carcinoma, cholangiocarcinoma (CCA) and mixed cell carcinoma, of which CCA is second to hepatocellular carcinoma (10%-15%) (3). Anatomically, CCA is a malignant tumor in the liver or bile duct, with biliary cell differentiation characteristics; including, into intrahepatic CCA (iCCA), perihilar CCA (pCCA), and distal CCA (dCCA) (4). Radical surgery is the most effective treatment of cholangiocarcinoma. About 30% of patients can receive radical surgery at the time of initial diagnosis, but the overall 5-year overall survival (OS) rate is less than 20% (5). Studies have reported that the main factors affecting the OS of CCA after radical surgery include: the status of surgical margins whether lymph nodes have metastasized, vascular invasion and normal liver tissue volume (6, 7). There are limited studies on the relationship between CCA and adjuvant chemotherapy (single drug, doublet drugs), recurrence pattern (local, regional, distant recurrence) and prognosis [(Disease free survival, DFS), OS] after radical surgery (R0 or R1 resection). Therefore, this study focuses on the correlation between CCA adjuvant chemotherapy regimen[single drug, mainly 5-fluorouracil (S-1 or capecitabine) adjuvant chemotherapy was administrated as follow:S-1 50 mg/m^2^ or capecitabine 1250 mg/m^2^ administered orally twice daily on days 1–14 for a 3-week cycle), doublet drugs, mainly gemcitabine + capecitabine or S-1 adjuvant chemotherapy was administrated as follow: gemcitabine 1000 mg/m^2^ administered intravenously on day 1 and S-1 50 mg/m^2^ or capecitabine 1000 mg/m^2^ administered orally twice daily on days 1–14 for a 3-week cycle], recurrence pattern and prognosis. All patients included in this study received S1/capecitabine or gemcitabine+S1/capecitabine regimen. The single drug regimen was received in oral S1/capecitabine for at least 4 cycles, and the doublet regimen was gemcitabine +S1/capecitabine for at least 2 cycles, and the dose should be adjusted according to toxic and side effects.

Retrospective analysis of preoperative hematological, clinical pathology, adjuvant chemotherapy regimen, recurrence pattern, DFS and OS data of 207 patients with CCA, was done. They were diagnosed by radical surgery, at the Chinese PLA General Hospital in the past 10 years. Chi-square test was used to analyze the correlation between related factors and postoperative recurrence. Survival curves were plotted by Kaplan-Meier method, P-values were calculated by Log-rank for DFS and OS univariate analysis, multivariate COX regression method was used for multivariate analysis.

## Materials and Methods

### Patient Selection

Retrospective analysis of clinical pathology data of patients with CCA in the Chinese PLA General Hospital, between October 2009 and March 2019 was done. The inclusion criteria were as follows: (1) Surgical treatment of primary CCA (The surgical strategy was to achieve complete microscopic clearance of the disease, including liver resection. Patients with less than 1 mm clearance were classified as surgical margin positive (R1) patients.), (2) Pathological diagnosis of CCA, (3) Complete admission and treatment information in the Chinese PLA General Hospital, with complete follow-up data available. The exclusion criteria were as follows: (1) Combine other cancer history. (2) Hepatocellular carcinoma or mixed cell carcinoma (including hepatocellular carcinoma and CCA). (3) Cases with unclear clinical or pathological stage. The judging criteria were as follows: (1) Pathological diagnostic criteria: According to the 4th edition of the WHO Classification of Digestive System Tumors, issued by the International Agency for Research on Cancer (IARC) in 2010.(2) Staging criteria: TNM staging for all patients with CCA according to the 8th edition of the Union for International Cancer Control (UICC). All patients were followed up after completion of treatment at a pre-defined frequency of once every 3 months in the first 2 years, once every 6 months from the third to the fifth year, and once yearly thereafter. Relapse or metastasis of the disease was diagnosed on contrast-enhanced magnetic resonance imaging or computed tomography.

### Data Collection

Data was obtained from medical records of CCA patients, retrospectively including: sex, age, white blood cell (WBC), hemoglobin (Hb), albumin (ALB), alpha fetoprotein(AFP), carcinoembryonic antigen(CEA), carbohydrate antigen 19-9(CA19-9), tumor number, tumor size, vascular invasion, pathological type, differentiation degree, TNM stage, adjuvant chemotherapy regimen, and recurrence pattern. This enabled us to explore the relationship between prognosis (DFS, OS) and adjuvant chemotherapy [single drug, mainly 5-fluorouracil (S-1 or capecitabine), and doublet gemcitabine based regimen], recurrence pattern [local, regional (within regional lymph node drainage), and distant recurrence (metastasis of other organs or lymph nodes beyond the scope of regional lymph node drainage)]. This study used telephone follow-up and inpatient medical records; the last follow-up was on March 1, 2020. The primary endpoint was OS and DFS; OS defined as the time from tumor diagnosis to follow-up or death, and DFS defined as the time from tumor diagnosis to disease recurrence. Death, last follow-up, and survival time were calculated in months.

### Statistical Methods

Laboratory data was collected from blood, routine liver function and tumor marker tests within 7 days to surgery. All data were analyzed using SPSS 24.0 statistical software. The Kaplan-Meier method was used to calculate the survival curve, the Log-rank method the P value of univariate analysis, and Chi-square test to analyze the correlation between related factors and postoperative recurrence. The forward stepwise method performed multivariate analysis of meaningful variables in a multivariate COX regression model; also calculated hazard ratio (HR) and 95% confidence interval (CI). All statistical tests were bilateral, the test level was α=0.05, and P<0.05 was considered statistically significant.

## Results

### General Characteristics

A total of 207 cases of CCA were reviewed; 135 (65.2%) male, 72 (34.8%) female, 114 (55.1%) less than 60 years old, and 93 (44.9%) were older than or equal to 60 years old. The median age was 59.0 years (31-88 years). 55 (26.6%) were in stage I and 108 (52.2%) in stage II. Sixty-three patients received adjuvant chemotherapy after surgery (15 single drug and 48 doublet drugs chemotherapy). Of the total, 156 cases (75.4%) recurred after the follow-up cut off time: 78 local, 40 regional and 38 distant recurrence cases. Median DFS was 14.0 months (2.1-119.0 months), whereas median OS was 22.1 months (3.9-119 months).

### Chi-Square Test Results of the Correlation Between Clinical Pathological Factors and Postoperative Recurrence

CCA patients (207) were divided into non-recurrence (51 cases) and recurrence (156 cases) groups. Chi-square test was used to analyze the correlation between relevant factors and postoperative recurrence. A total of 15 factors were included for group analysis (**Table 1**). The statistically significant factors were: CA19-9 (≥35 vs < 35,p=0.04), vascular invasion (p=0.04), and adjuvant chemotherapy (Single drug vs Doublet drugs,p=0.02).

**Table 1:** Chi-square test results of the correlation between the clinical pathological factors and recurrence of cholangiocarcinoma after radical surgery.

Variable	No-recurrence group [cases(%)]	Recurrence group [cases(%)]	*P* value
Sex			
Female	17 (33.3)	55 (35.3)	0.80
Male	34 (66.7)	101 (64.7)	
Age (years)			
<60	30 (58.8)	84 (53.8)	0.54
≥60	21 (41.2)	72 (46.2)	
WBC (10^9/L)			
<10	45 (88.2)	138 (88.5)	0.97
≥10	6 (11.8)	18 (11.5)	
HB (g/L)			
<120	16 (31.4)	50 (32.1)	0.93
≥120	35 (68.6)	106 (67.9)	
ALB (g/L)			
<35	13 (25.5)	42 (26.9)	0.84
≥35	38 (74.5)	114 (73.1)	
AFP (ug/L)			
<10	50 (98.0)	149 (95.5)	0.42
≥10	1 (2.0)	7 (4.5)	
CEA (ug/L)			
<5	43 (84.3)	132 (84.6)	0.96
≥5	8 (15.7)	24 (15.4)	
CA19-9 (U/ml)			
<35	20 (39.2)	38 (24.4)	0.04
≥35	31 (60.8)	118 (75.6)	
Tumor number			
Single	48 (94.1)	147 (94.2)	0.98
Multiple	3 (5.9)	9 (5.8)	
Tumor size(cm)			
<5	44 (86.3)	128 (82.1)	0.49
≥5	7 (13.7)	28 (17.9)	
Vascular invasion			
No	48 (94.1)	128 (82.1)	0.04
Yes	3 (5.9)	28 (17.9)	
Pathological type			
Intrahepatic	10 (19.6)	33 (21.1)	0.87
Perihilar	5 (29.4)	50 (32.1)	
Distal	26 (51.0)	73 (46.8)	
Differentiation degree			
Poor	15 (29.4)	63 (40.4)	0.37
Moderate	31 (60.8)	81 (51.9)	
Well	5 (9.8)	12 (7.7)	
TNM stage			
I	18 (35.3)	37 (23.7)	0.22
II	22 (43.1)	86 (55.1)	
III	11 (21.6)	33 (21.2)	
Adjuvant chemotherapy			
Single drug	1 (5)	14 (32.6)	0.02
Doublet drugs	19 (95)	29 (67.4)	

WBC, white blood cell; HB, hemoglobin; ALB, albumin; AFP, alpha fetoprotein; CEA, carcinoembryonic antigen; CA19-9, carbohydrate antigen 19-9; Single drug, mainly 5-fluorouracil (S-1 or capecitabine) adjuvant chemotherapy was administrated as follow:S-1 50 mg/m^2^ or capecitabine 1250 mg/m^2^ administered orally twice daily on days 1–14 for a 3-week cycle; Doublet drugs, mainly gemcitabine + capecitabine or S-1 adjuvant chemotherapy was administrated as follow: gemcitabine 1000 mg/m^2^ administered intravenously on day 1 and S-1 50 mg/m^2^ or capecitabine 1000 mg/m^2^ administered orally twice daily on days 1–14 for a 3-week cycle.

### DFS and OS Univariate Analysis Results

The data of 207 cases of CCA were analyzed by Kaplan-Meier method whereas the P-value of Log-rank was analyzed by univariate analysis. A total of 14 clinical pathological factors were included (**Table 2**). DFS univariate analysis showed statistical differences among CA19-9 (p=0.04), vascular invasion (p=0.00), differentiation degree (p=0.01), TNM stage (p=0.03) and adjuvant chemotherapy (p=0.01). OS univariate factor analysis results showed that CA19-9 (p=0.01), vascular invasion (p=0.00), differentiation degree (p= 0.01), TNM stage (p=0.01) and recurrence pattern (p=0.01) were statistically significant.

**Table 2:** Results of univariate analysis of disease-free survival and overall survival after cholangiocarcinoma radical surgery.

Variable	Grouping	Disease-free survival		Overall survival	
		Median OS (month)	*P* value	Median OS (month)	*P* value
WBC (10^9/L)	<10	23.8	0.99	32.0	0.85
	≥10	25.8		36.1	
Hb (g/L)	<120	30.3	0.93	35.2	0.92
	≥120	23.9		32.0	
ALB (g/L)	<35	14.3	0.17	27.8	0.29
	≥35	24.6		32.3	
AFP (ug/L)	<10	24.0	0.46	35.0	0.91
	≥10	16.2		25.1	
CEA (ug/L)	<5	24.0	0.62	35.2	0.35
	≥5	19.9		27.8	
CA19-9 (U/ml)	<35	41.1	0.04	53.7	0.01
	≥35	22.0		28.6	
Tumor number	Single	23.9	0.95	32	0.67
	Multiple	38.5		47.8	
Tumor size(cm)	<5	25.8	0.13	32.3	0.92
	≥5	15.6		32.0	
Vascular invasion	No	27.3	0.00	36.1	0.00
	Yes	8.6		16.2	
Pathological type	Intrahepatic	15.1	0.13	25.1	0.15
	Perihilar	23.8		32.0	
	Distal	31.8		42.0	
Differentiation degree	Poor	16.2	0.01	24.0	0.01
	Moderate	27.3		35.2	
	Well	41.8		65.4	
TNM stage	I	47.3	0.03	54.0	0.01
	II	20.1		28.6	
	III	17.6		27.8	
Adjuvant chemotherapy	Single drug	11.0	0.01	27.1	0.20
	Doublet drugs	24.6		31.3	
Recurrence patterns	Local			31.2	0.01
	Regional			32.0	
	Distant			20.4	

WBC, white blood cell; Hb, hemoglobin; ALB, albumin; AFP, alpha fetoprotein; CEA, carcinoembryonic antigen; CA19-9, carbohydrate antigen 19-9; Single drug, mainly 5-fluorouracil (S-1 or capecitabine) adjuvant chemotherapy was administrated as follow:S-1 50 mg/m^2^ or capecitabine 1250 mg/m^2^ administered orally twice daily on days 1–14 for a 3-week cycle; Doublet drugs, mainly gemcitabine + capecitabine or S-1 adjuvant chemotherapy was administrated as follow: gemcitabine 1000 mg/m^2^ administered intravenously on day 1 and S-1 50 mg/m^2^ or capecitabine 1000 mg/m^2^ administered orally twice daily on days 1–14 for a 3-week cycle.

### DFS and OS Multivariate Analysis Results

**Figure 1:**
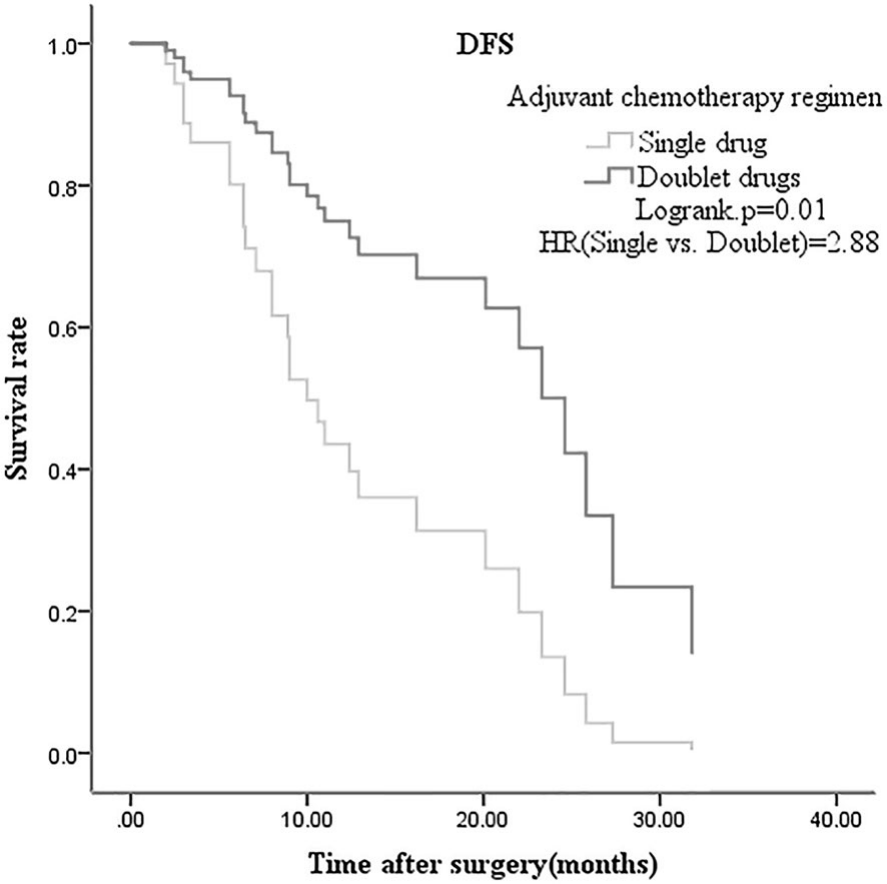
Disease free survival multivariate analysis of radical postoperative cholangiocarcinoma grouped by adjuvant chemotherapy.

**Figure 2:**
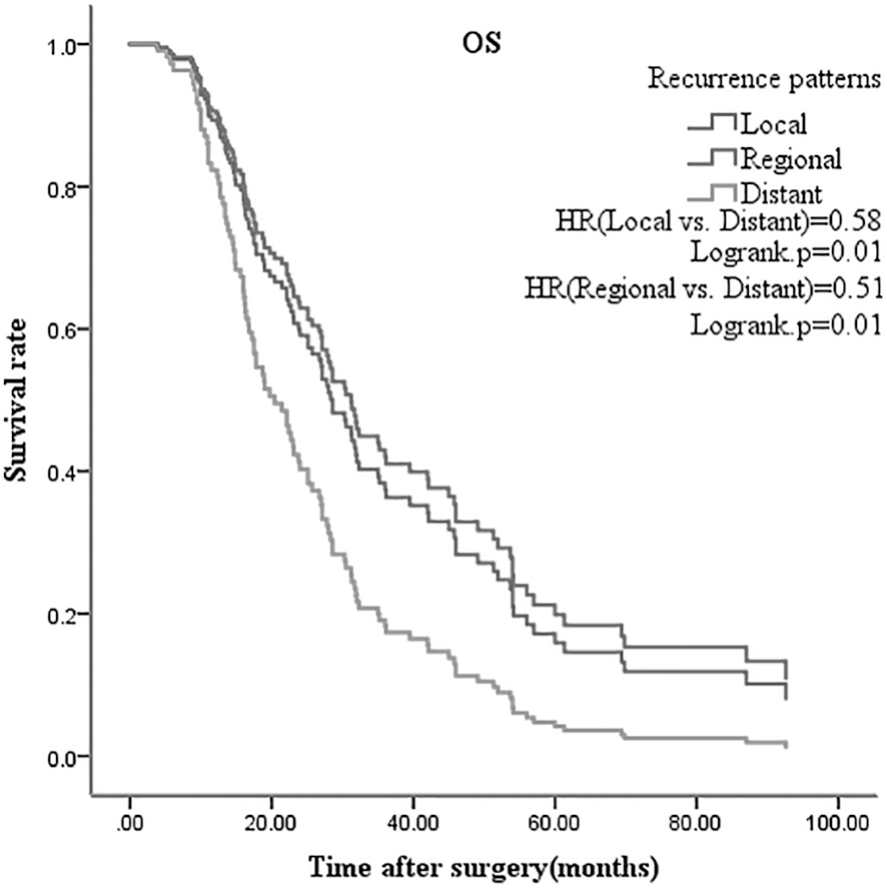
Overall survival multivariate analysis of radical cholangiocarcinoma surgery according to recurrence pattern.

Multivariate analysis of meaningful variables in multivariate COX regression model was performed using forward stepwise method; variables with p<0.05 were included in the model (CA19-9, vascular invasion, differentiation degree, TNM stage, adjuvant chemotherapy, recurrence pattern). DFS analysis showed that vascular invasion (p=0.02) and adjuvant chemotherapy (p=0.01) were independent prognostic factors of CCA, after radical surgery. OS analysis showed that CA19-9 (p=0.02), vascular invasion (p=0.01), and recurrence pattern (p=0.01) were independent prognostic factors (**Tables 3**, **4**). Adjuvant chemotherapy (gemcitabine based doublet drug regimen) and recurrence pattern (local and regional) have better prognosis (**Figures 1**, **2**).

**Table 3:** Multivariate analysis of disease-free survival after radical cholangiocarcinoma surgery.

Variable	HR (95%CI)	*P* value
Vascular invasion (no vs. yes)	0.35 (0.14-0.86)	0.02
Adjuvant chemotherapy (Single drug vs. Doublet drugs)	2.88 (1.33-6.25)	0.01

Single drug, mainly 5-fluorouracil (S-1 or capecitabine) adjuvant chemotherapy was administrated as follow:S-1 50 mg/m^2^ or capecitabine 1250 mg/m^2^ administered orally twice daily on days 1–14 for a 3-week cycle; Doublet drugs, mainly gemcitabine + capecitabine or S-1 adjuvant chemotherapy was administrated as follow: gemcitabine 1000 mg/m^2^ administered intravenously on day 1 and S-1 50 mg/m^2^ or capecitabine 1000 mg/m^2^ administered orally twice daily on days 1–14 for a 3-week cycle.

**Table 4:** Multivariate analysis of overall survival after radical cholangiocarcinoma surgery.

Variable	HR (95%CI)	*P* value
CA19-9 (<35 vs. ≥35)	0.59 (0.37-0.92)	0.02
Vascular invasion (no vs. yes)	0.49 (0.30-0.79)	0.01
Recurrence patterns (local vs. distant)		0.02
	0.58 (0.37-0.89)	0.01
(regional vs. distant)	0.51 (0.30-0.86)	0.01

CA19-9, carbohydrate antigen 19-9.

## Discussion

Cholangiocarcinoma (CCA) usually has no obvious early stage symptoms, and most cases are diagnosed at a later stage; by tumor markers, imaging and pathological examination (8). Few patients accept radical surgery and liver transplantation on late stage diagnosis. Most patients show disease recurrence and progression, even after undergoing radical surgery (9). There are limited treatment options for advanced CCA; chemotherapy is not efficient and targeted drugs have not proven to be beneficial, in high level evidence based medicine (10, 11).In our study, middle-aged population than in the elderly (55.1% vs. 44.9%), and females than men (65.2% vs. 34.8%). In addition, of the 152 (73.4%) postoperative TNM stage II and III patients, the overall 1-year DFS population rate was 58.0%, and the 2-year OS rate was 46.4%.

In this study, 207 cases of CCA were divided into non-recurrence (51 cases) and recurrence (156 cases) groups. Chi-square test showed that high CA19-9 (≥35U/ml), vascular invasion, and single drug adjuvant chemotherapy are associated with postoperative recurrence (p<0.05). DFS and OS (p<0.05 in the univariate analysis) were included in the multivariate analysis of the COX regression model. It was found that vascular invasion and adjuvant chemotherapy were independent prognostic factors of DFS, whereas CA19-9, vascular invasion and recurrence patterns for OS. In radical surgery, most patients still showed disease recurrence and progression. Patel et al. (12) found that the preoperative CA19-9 value for diagnosing intrahepatic CCA had sensitivity 62% and specificity of 63%; and the overall CA19-9 value was higher in inoperable than in operable patients. Tamandl et al. (13) found that preoperative CA19-9 value less than 100U/ml is associated with better DFS. Zhu Y et al. (14) suggests the preoperative CA19-9 can be used to determine postoperative recurrence, besides being a diagnostic indicator. In our study, the preoperative CA19-9 (≥35U/ml) was found to be related to postoperative recurrence and an independent prognostic factor affecting OS. Wang C et al. (15) analyzed the risk factors of early (<12 months) and late recurrence (≥12 months) of intrahepatic CCA after radical surgery, and found that vascular invasion and high preoperative CA19-9 value were independent risk factors for early recurrence. In our research 90.3% of patients with vascular invasion (28/31) experienced recurrence after radical surgery, and it was also an independent prognostic factor of DFS and OS (no vascular invasion vs. vascular invasion, HR=0.35 and HR=0.59, respectively). It has been confirmed that vascular invasion is a risk factor for postoperative recurrence (7), but there are few reports on whether it affects OS. In our study, vascular invasion caused poor OS of CCA patients; this may be related to lack of effective treatment after the recurrence. Choi SB et al. (6) found that the degree of differentiation is an independent prognostic factor for postoperative DFS of intrahepatic CCA, but this is controversial. In our study, the degree of differentiation was not related to postoperative recurrence, and also not statistically significant in DFS and OS, on multivariate analysis. Our research included intrahepatic, perihilar and distal CCA, and 43 intrahepatic cases presented as poorly and moderately differentiated; this may reduce the differences between groups and affect the results. Valle J et al. (16) confirmed that the combined drug regimen, gemcitabine with cisplatin, is the better first-line standard therapy and can bring survival benefits for advanced CCA; however, there is controversy about its value after radical surgery. The BILCAP study was designed to explore whether capecitabine adjuvant therapy brings survival benefits, as compared to placebo. Subgroup analysis showed that adjuvant chemotherapy prolongs OS for poor differentiated CCA (17). Our study found median DFS of 11.0 months for 15 patients on single-agent adjuvant chemotherapy (oral capecitabine or S-1), and 24.6 months for 48 patients with doublet agents (gemcitabine + capecitabine or S-1). The difference in mDFS between our study and BILCAP may be related to the following factors:1) The gallbladder cancer and distal cholangiocarcinoma patients accounted for 52.6% (235/447) in BILCAP study, while our study included intrahepatic and hilar cholangiocarcinoma. Previous studies have shown that gallbladder and distal cholangiocarcinoma harbor a better prognosis, while intrahepatic and hilar cholangiocarcinoma were prone to recurrence.2) In the BILCAP study, stage III accounted for 10.3% (46/447), while 21.3% (44/201) were included in this study. Higher proportion of stage III may affect the results of DFS. Adjuvant chemotherapy (single vs. double regimen) is an independent prognostic factor for DFS (HR: 2.88, p=0.01). The following factors may affect the results of our study: 1.) Retrospective studies have selection bias, and the small number of single drug subgroups can affect the differences between groups; 2.) It seems that oral maintenance therapy after doublet drugs adjuvant chemotherapy can improve DFS; 47.9% (23/48) of the subgroup received oral maintenance therapy (S-1 or capecitabine). Yu W et al. (18) retrospectively studied 73 cases of postoperative intrahepatic CCA recurrence pattern and found that regional lymph nodes were the most commonly affected site; but there was limited data on the impact of recurrence pattern on prognosis. Our research indicates that the most common site of recurrence is local (78/156), with the pattern being an independent prognostic factor of OS. However, this study did not analyze the effectiveness of treatment after recurrence and its impact on progression free survival (PFS) and OS. Biliary malignant tumor were characterized by various gene mutations, strong heterogeneity and poor prognosis. Future clinical studies may require combination with molecular pathological and genetic testing. Our team will explore this further in subsequent studies.

This study focuses on the correlation between CCA, adjuvant chemotherapy regimen, recurrence pattern and prognosis, after radical surgery. However, the study involves a single-center retrospective analysis with a large time span, and the variables were collected at a single time point before surgery, and no dynamic change in data was obtained, most of the patients who did not receive postoperative adjuvant chemotherapy were stage I patients, and the postoperative DFS and OS were longer. We were concerned that the inclusion of these patients would affect the overall analysis results, so we did not include, this may affect the final conclusion of the study. Retrospective analysis of 207 CCA cases of radical surgery showed that: 1.) Preoperative higher CA19-9 value, vascular invasion, and single drug adjuvant chemotherapy were associated with postoperative recurrence; 2.) Vascular invasion and adjuvant chemotherapy were the independent prognostic factors of DFS, 3.) Preoperative CA19-9 value, vascular invasion and recurrence pattern were independent prognostic factors of OS. Our study showed that for radical surgical cholangiocarcinoma, For those patients with high risk factors for postoperative recurrence (such as R1 resection, Vascular invasion, stage III and high CA199 value), Adjuvant chemotherapy with doublet drug regimen is correlated with longer DFS, and different recurrence modes affect OS.

## Data Availability Statement

The raw data supporting the conclusions of this article will be made available by the authors, without undue reservation.

## Ethics Statement

This study involving human participants were reviewed and approved by the ethics committee of Chinese PLA General Hospital. Written informed consent to participate in this study was waivered by the ethics committee.

## Author Contributions

BQ: Conception and design. LD: Administrative support. LR: Data analysis and interpretation. NM: Provision of study materials and methods. YL: Collection and assembly of data. All authors contributed to the article and approved the submitted version.

## Conflict of Interest

The authors declare that the research was conducted in the absence of any commercial or financial relationships that could be construed as a potential conflict of interest.

## Publisher’s Note

All claims expressed in this article are solely those of the authors and do not necessarily represent those of their affiliated organizations, or those of the publisher, the editors and the reviewers. Any product that may be evaluated in this article, or claim that may be made by its manufacturer, is not guaranteed or endorsed by the publisher.
